# Therapeutic Synergy Between Antibiotics and Pulmonary Toll-Like Receptor 5 Stimulation in Antibiotic-Sensitive or -Resistant Pneumonia

**DOI:** 10.3389/fimmu.2019.00723

**Published:** 2019-04-09

**Authors:** Laura Matarazzo, Fiordiligie Casilag, Rémi Porte, Frederic Wallet, Delphine Cayet, Christelle Faveeuw, Christophe Carnoy, Jean-Claude Sirard

**Affiliations:** Univ. Lille, CNRS, Inserm, CHU Lille, Institut Pasteur de Lille, U1019 - UMR8204 - CIIL - Center for Infection and Immunity of Lille, Lille, France

**Keywords:** flagellin, Toll-like receptor 5, antibiotic, resistance, *Streptococcus pneumoniae*, pneumonia, superinfection

## Abstract

Bacterial infections of the respiratory tract constitute a major cause of death worldwide. Given the constant rise in bacterial resistance to antibiotics, treatment failure is increasingly frequent. In this context, innovative therapeutic strategies are urgently needed. Stimulation of innate immune cells in the respiratory tract [via activation of Toll-like receptors (TLRs)] is an attractive approach for rapidly activating the body's immune defenses against a broad spectrum of microorganisms. Previous studies of the TLR5 agonist flagellin in animal models showed that standalone TLR stimulation does not result in the effective treatment of pneumococcal respiratory infection but does significantly improve the therapeutic outcome of concomitant antibiotic treatment. Here, we investigated the antibacterial interaction between antibiotic and intranasal flagellin in a mouse model of pneumococcal respiratory infection. Using various doses of orally administered amoxicillin or systemically administered cotrimoxazole, we found that the intranasal instillation of flagellin (a dose that promotes maximal lung pro-inflammatory responses) induces synergistic rather than additive antibacterial effects against antibiotic–susceptible pneumococcus. We next set up a model of infection with pneumococcus that is resistant to multiple antibiotics in the context of influenza superinfection. Remarkably, the combination of amoxicillin and flagellin effectively treated superinfection with the amoxicillin-resistant pneumococcus since the bacterial clearance was increased by more than 100-fold compared to standalone treatments. Our results also showed that, in response to flagellin, the lung tissue generated an innate immune response even though it had been damaged by the influenza virus and pneumococcal infections. In conclusion, we demonstrated that the selective boosting of lung innate immunity is a conceptually advantageous approach for improving the effectiveness of antibiotic treatment and fighting antibiotic-resistant bacteria.

## Introduction

Pneumonia constitutes a major cause of death, morbidity and health resource use worldwide. The main causative agents identified in adult patients hospitalized for community-acquired pneumonia (CAP) are viruses (in 27–30% of cases, the most common being rhinovirus, influenza and coronavirus) and bacteria (14–23% of cases, with a marked predominance of *Streptococcus pneumoniae* infections) (1–3). When faced with overt clinical signs of bacterial pneumonia, the standard of care is antibiotic treatment. The combination of a constant rise in antibiotic resistance in recent decades with a decline in the discovery of new drugs has led to an increase in treatment failure and mortality (4). In 2017, the World Health Organization's Global Action Plan highlighted the urgent need to control the emergence of antibiotic resistance (5). Given this context, a number of new anti-infectious treatment strategies are being developed.

The modulation of innate immunity [by targeting immune receptors, such as Toll-like receptors (TLRs)] is a promising approach (6, 7). Indeed, innate immunity is highly conserved in evolution, and this system constitutes the first line of defense against invading pathogens. Moreover, innate immunity triggers a broad range of antimicrobial defense mechanisms and immune cells—thereby greatly reducing the risk of resistance in the pathogens. Moreover, activation of TLR signaling has been associated with a favorable outcome in infections with antibiotic-resistant bacteria or colonization resistance by such pathogens (8–10). These observations support that stimulation and effector activities of innate immunity are not influenced by the antibiotic resistance mechanisms carried by bacteria. Flagellin is the main protein component of the bacterial flagellum and is a natural agonist of TLR5; the latter is expressed at the surface of a many different cell types, including mucosal epithelial cells and immune cells such as dendritic cells, macrophages, and lymphocytes (11). Various studies in animal models have highlighted the antimicrobial potency of flagellin against a wide variety of bacterial infections [such as intestinal infections caused by *Salmonella enterica, Enterococcus faecium, Clostridium difficile*, and *Escherichia coli* (8, 12–14), respiratory infections caused by *Pseudomonas aeruginosa* and *S. pneumoniae* (15, 16)], and viral and fungal infections (17–19). Although most studies have demonstrated the protective effect of flagellin administered before or during exposure to a microbial pathogen, the protein's immunostimulatory efficacy in therapeutic context has not been extensively characterized. Using a mouse model of *S. pneumoniae* lung infection, we recently demonstrated that combination treatment with mucosally administered flagellin and an orally or intraperitoneally administered low-dose (i.e., subtherapeutic) antibiotic is more effective than the antibiotic alone (i.e., with a lower bacterial load in the lung, and a lower mortality rate). Furthermore, the combination treatment was also effective in a model of post-flu pneumococcal superinfection (20). The effectiveness of these combination therapies depends on TLR5 signaling as demonstrated using TLR5-deficient animals and TLR5-mutated recombinant flagellin (20). Our studies highlighted that the airway epithelium is the main TLR5-specific signaling compartment (21–23). Taken as a whole, these observations are the first to highlight the added value of respiratory delivery of flagellin as an immunomodulatory biologic for the adjunct treatment of bacterial pneumonia (i.e., in addition to the standard of care).

Our working hypothesis was that simultaneous treatment with an antibiotic and intranasal, i.e., respiratory flagellin constitutes a “double hit” against the pathogen. A combination of two drugs may result in independent actions or specific (i.e., additive, synergistic, or antagonistic) effects that define the biological outcome (24–26). An interaction between two drugs is considered to be synergistic when the measured effect of the combination treatment exceeds the predicted cumulative value of the two components given separately. Synergy increases treatment efficacy, and is expected to limit the emergence of drug resistance. Furthermore, synergy allows the physician to decrease the dose level or the frequency of dosing, which thereby dampens adverse drug reactions and may even enable the rehabilitation of neglected drugs. Conversely, an antagonistic combination treatment has a smaller effect than the predicted cumulative value of the two components given separately. Most studies of potentially synergistic antimicrobial agents are performed in *in vitro* systems such as bacterial cultures, using checkerboard assays and increasing doses of each drug (25, 27). Unlike antibiotics that directly affect the bacteria, immunomodulatory biologic activity requires sentinel cells for detection, downstream signaling and thus the production of antimicrobial effectors and the recruitment and/or activation of innate immune cells. At present, there are no comprehensive *in vitro* models of this complicated physiological system.

In the present study, we quantified the nature and magnitude of the interactions between antibiotics and intranasal instillation of flagellin with regard to antibacterial effectiveness in a murine model of *S. pneumoniae* respiratory infections. Furthermore, we wanted to assess the efficacy of this novel therapeutic strategy against infection with antibiotic-resistant bacteria, which represents major public health issues today. To this aim, we investigated the combination's effect on antibiotic-resistant *S. pneumoniae* in a relevant model of post-flu pneumococcal pneumonia, and characterized the immune response induced by the flagellin-mediated protection.

## Materials and Methods

### Bacterial Strains and Cultures

Serotype 1 *S. pneumoniae* (Sp1; clinical isolate E1586) was obtained from the National Reference Laboratory—Ministry of Health, Uruguay (15). Serotype 3 *S. pneumoniae* (Sp3; strain 104491) was provided by the Institut Pasteur (Paris, France); it is a multidrug-resistant clinical isolate from a human bronchial secretion, and is resistant to amoxicillin (AMX), cefotaxime, doxycycline, erythromycin, chloramphenicol, streptomycin, and cotrimoxazole (SXT). Working stocks were prepared as described previously (15, 28). Briefly, fresh colonies grown on blood-agar plates were incubated in Todd Hewitt Yeast Broth (THYB) (Sigma-Aldrich, Saint-Louis, MO) at 37°C until the OD_600nm_ reached 0.7–0.9 units. Cultures were stored at −80°C in THYB + glycerol 12% (vol./vol.) for up to 3 months. For infection, working stocks were thawed and washed with sterile Dulbecco's Phosphate-Buffered Saline (PBS, Gibco, Grand Island, NY) and diluted to the appropriate concentration. The number of bacteria (as colony forming units [CFUs]) was confirmed by plating serial dilutions onto 5% sheep blood agar plates.

### Mouse Models of Infection

Female BALB/cJ mice, female Swiss mice, and male C57BL/6J mice (6–8 weeks old) (Janvier Laboratories, Saint Berthevin, France, or Envigo, Huntingdon, UK) were maintained in individually ventilated cages and handled in a vertical laminar flow cabinet (class II A2, ESCO, Hatboro, PA). All experiments complied with institutional regulations and ethical guidelines (C59-350009, Institut Pasteur de Lille; Protocol 2015121722429127). Prior to intranasal infection, the mice were anesthetized via the intraperitoneal injection of 1.25 mg (50 mg/kg) ketamine plus 0.25 mg (10 mg/kg) xylazine in 250 μl of PBS. For primary infections with Sp1, 2–4 × 10^6^ CFU were inoculated intranasally in 30 μl PBS, as described previously (20). The influenza infection model was developed in our laboratory on the C57BL/6J mice (29, 30). The Sp3 pneumococcal superinfection model was therefore performed in these animals. Briefly, mice were first infected intranasally with 30 μl PBS containing 50 plaque-forming units (PFUs) of the pathogenic, murine-adapted H3N2 influenza A virus strain Scotland/20/74, as described previously (30, 31). Seven days later, animals were infected intranasally with 10^3^ CFU of Sp3 in 30 μl PBS. For the determination of bacterial counts in lung and spleen, mice were sacrificed at selected times via the intraperitoneal injection of 5.47 mg of sodium pentobarbital in 100 μl PBS. Tissues were collected and homogenized with an UltraTurrax homogenizer (IKA-Werke, Staufen, Germany), and viable counts were determined by plating serial dilutions onto blood agar plates and incubating them at 37°C for 12–24 h.

### Flagellin and Antibiotic Administration

The recombinant flagellin FliC_Δ174−400_ came from *S. enterica* serovar Typhimurium FliC and was produced with an histidine tag, as described previously (20, 32). The protein FliC_Δ174−400_ was certified to be immunologically active in reporter cells and in mouse assays, and the residual lipopolysaccharide concentration was determined to be < 20 pg per μg of flagellin (20). For flagellin treatment, FliC_Δ174−400_ (1 ng to 25 μg in 30 μl PBS) was administrated intranasally under light anesthesia via isoflurane inhalation (Axience, Pantin, France). Control animals received intranasal PBS alone. Mice were treated either intragastrically with AMX [5–350 μg of amoxicillin trihydrate (Sigma-Aldrich) in 200 μl water per animal] or intraperitoneally with SXT—a combination of the antibiotics sulfamethoxazole and trimethoprim (Bactrim® Roche, Basel, Switzerland) at total doses of 1 mg (0.84 mg sulfamethoxazole and 0.16 mg trimethoprim) or 4 mg (3.34 mg sulfamethoxazole and 0.66 mg trimethoprim) in 200 μl PBS per animal.

### Testing for Synergy and Proportional Effects

The treatments' effects on *S. pneumoniae* lung infection were quantified as the percentage bacterial growth (%_growth_), corresponding to the ratio of the mean bacterial load in the lungs of infected, treated mice to the load in infected, non-treated (control) mice. For example, the effect of treatment A was calculated as follows: %_growth[A]_ = (mean CFU_[A]_/mean CFU_[control]_) × 100. The predicted additive effect (or predicted %_growth_) of a combination treatment was calculated as described previously (33). Briefly, the predicted %_growth_ of a treatment combining compounds A and B is the product of the experimentally defined %_growth_ values for each standalone treatment (predicted%_growth[A+B]_ = %_growth[A]_ × %_growth[B]_). If the experimental %_growth_ for the combination treatment is lower or higher than the predicted %_growth_, then the two drugs are synergistic or antagonistic, respectively. When the experimental and predicted %_growth_ values are identical, the two drugs' effects are additive.

### Transcriptional Analysis by RT-qPCR

Total lung RNA was extracted with the NucleoSpin RNA Plus kit (Macherey-Nagel, Duren, Germany) and reverse-transcribed with the High-Capacity cDNA Archive Kit (Applied Biosystems, Foster City, CA). The cDNA was amplified using SYBR green-based real-time PCR on a Quantstudio 12K PCR system (Applied Biosystems). Relative mRNA levels (2^−ΔΔCT^) were determined by comparing first the PCR cycle thresholds (C_q_) for the gene of interest and the reference genes *Actb* and *B2m* (ΔC_q_), and then the ΔC_q_ values for infected mice treated with the AMX+flagellin combination treatment and with AMX alone (control group) (ΔΔCq). All the primers used in the study (listed in Table 1) were validated for efficacy.

**Table 1:** Sequences of the primers used for qPCR assays.

**Target gene**	**Forward primer (F)**	**Reverse primer (R)**
*Actb*	CGTCATCCATGGCGAACTG	GCTTCTTTGCAGCTCCTTCGT
*B2m*	TGGTCTTTCTGGTGCTTGTC	GGGTGGCGTGAGTATACTTGAA
*Ccl20*	TTTTGGGATGGAATTGGACAC	TGCAGGTGAAGCCTTCAACC
*Cxcl1*	CTTGGTTCAGAAAATTGTCCAAAA	CAGGTGCCATCAGAGCAGTCT
*Cxcl2*	CCCTCAACGGAAGAACCAAA	CACATCAGGTACGATCCAGGC
*Il1b*	AATCTATACCTGTCCTGTGTAATGAAAGAC	TGGGTATTGCTTGGGATCCA
*Il6*	GTTCTCTGGGAAATCGTGGAAA	AAGTGCATCATCGTTGTTCATACA
*S100a9*	CACCCTGAGCAAGAAGGAAT	TGTCATTTATGAGGGCTTCATTT

### Cell Analysis by Flow Cytometry

Bronchoalveolar lavage (BAL) fluid samples were obtained after intratracheal injection of 3 × 1 ml of PBS supplemented with 5% fetal calf serum (FCS). Lungs were perfused with PBS, excised and finely minced then digested in a solution of RPMI 1640 medium (Gibco) containing 1 mg/ml collagenase VIII (Sigma-Aldrich) and 80 μg/ml DNase I (Sigma-Aldrich) for 20 min at 37°C. After washes, red blood cells were removed using a lysis solution (Pharmlyse, BD Bioscience). Lung cell homogenates were then suspended in a 20% percoll gradient and centrifuged at 2,000 rpm without brake at room temperature for 10 min. The cell pellets were washed with PBS supplemented with 2% FCS and cells were filtrated before antibody labeling. BAL and lung cells were stained with anti-CD45-allophycocyanin-cyanine 7 (clone 30F11), anti-CD11b-Brilliant Violet 785 (clone M1.70), anti-SiglecF-AlexaFluor 647 (clone E50-2440), anti-Ly6C-peridinin chlorophyll protein-cyanine 5.5 (clone HK1.4), anti- Ly6G-phycoerythrin (clone 1A8), anti-CD11c-phycoerythrin-cyanine 7 (clone HL3), and CCR2-Brillant Violet 421 (clone SA203G11) antibodies. Dead cells were excluded from the analysis using propidium iodide. The antibodies were purchased from BD Biosciences (San Jose, CA) or BioLegend (San Diego, CA). Data were collected on a BD LSR Fortessa and analyzed with the BD FACSDiva software.

### Cytokine and Chemokine Production

Concentration of CCL20, CXCL1, CXCL2, IL-6, IL-1β, and TNF was determined in BAL fluids and lung homogenates by enzyme-linked immunosorbent assay (ELISA kit from eBioscience, R&D Systems or Becton Dickinson). BAL fluids were obtained by intratracheal injection of 2 × 1 ml PBS supplemented with protease inhibitors (Roche). Lungs were perfused with PBS and collected in T-PER reagent (Pierce) supplemented with protease inhibitors and debris were eliminated by centrifugation. All samples were stored at −20°C.

### Statistical Analysis

The results were described as the mean ± standard error of the mean (SEM) or the median (range), as indicated. Intergroup differences were analyzed using the Mann-Whitney test and the log rank test. All analyses were performed with Prism software (version 5.0, GraphPad Software, La Jolla, CA). The threshold for statistical significance was set to *p* < 0.05.

## Results

### Determination of the Minimum Dose of Intranasal Flagellin for the Full Activation of Respiratory Tract Innate Immune Responses

**Figure 1:**
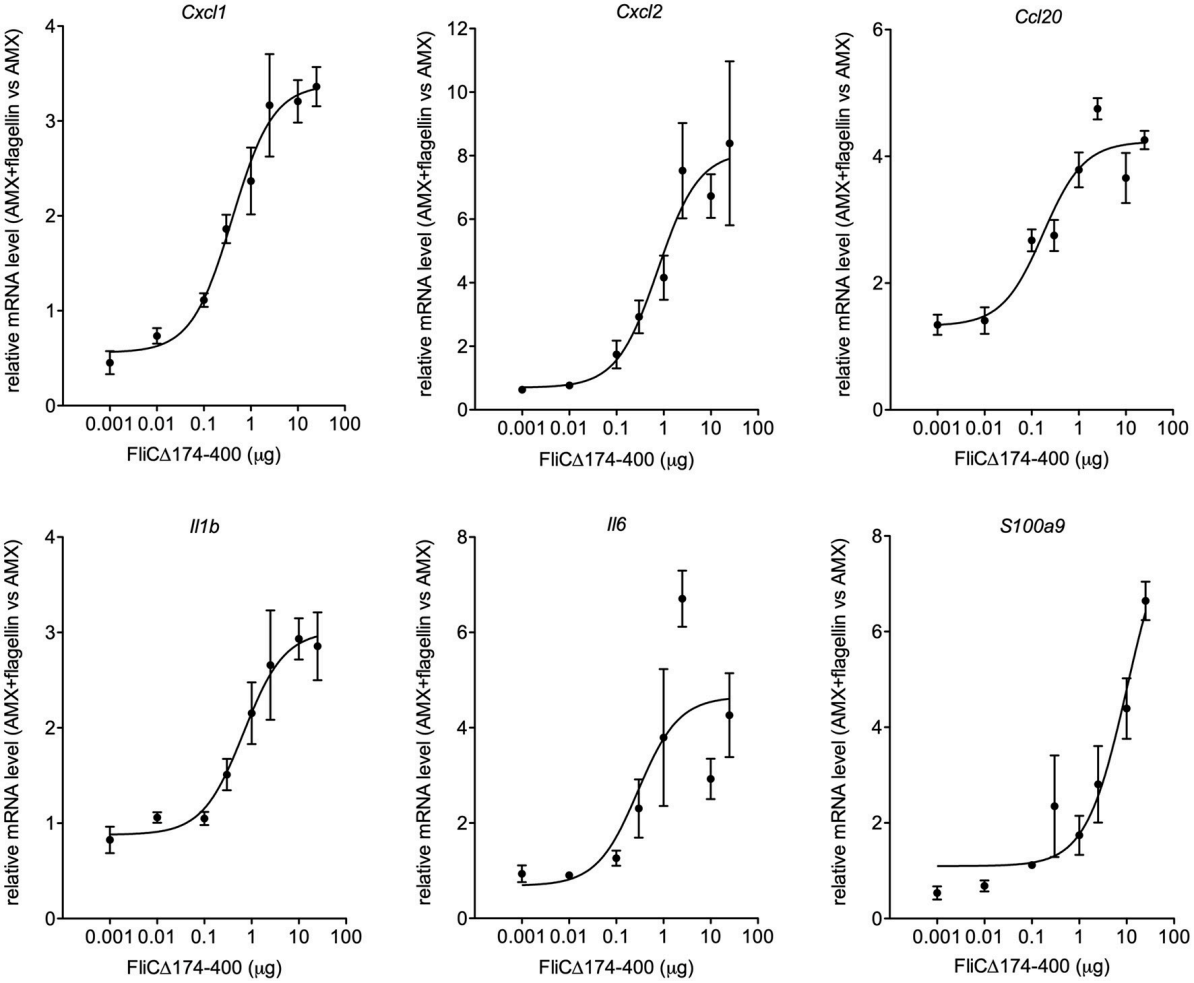
The effect of the flagellin dose on the transcriptional response of immune system-related genes. BALB/c mice (*n* = 4 per group) were infected intranasally with 2 × 10^6^ Sp1 and treated 12 h later with the antibiotic amoxicillin (AMX; 5 μg, intragastric administration) combined with the intranasal administration of various doses of flagellin FliC_Δ174−400_ (0.001, 0.1, 0.3, 1, 2.5, 10, and 25 μg in 30 μl of PBS) or vehicle only (PBS). Lungs were collected 2 h post-treatment, and RNA was extracted and reverse-transcribed. Gene expression was analyzed using quantitative PCR assays. The relative expression level for each gene is expressed against that of the reference genes *Actb* and *B2m* and the reference condition AMX+PBS (arbitrarily set to a value of 1). The data are quoted as the mean ± SEM.

In earlier research, we had shown that the intranasal administration of a combination of flagellin FliC_Δ174−400_ and low-dose antibiotics improved the therapeutic outcome of lung infection with the antibiotic-susceptible Sp1 [minimum inhibitory concentration (MIC)_AMX_ = 0.016 μg/ml] (20). Given the difficulty of performing *in vitro* checkerboard assays with immunomodulators, we therefore sought to evaluate the nature of antibiotic-flagellin interactions *in vivo*. We first defined the dose of flagellin that promoted saturating immune responses in Sp1-infected mice (Figure 1). Intranasally administered flagellin was associated with the production of various innate immunity-related components, including chemokines (CXCL1, CXCL2, and CCL20), inflammatory cytokines (IL-1β and IL-6), and antimicrobial peptides (S100A9), along with the recruitment of neutrophils to the airways (15, 16, 20, 21, 23, 28). Mice were treated simultaneously with oral AMX (0.2 mg/kg) and intranasal flagellin FliC_Δ174−400_ (at doses of 0.4 μg to 1 mg/kg, i.e., 1 ng to 25 μg per animal). Immune responses were analyzed by monitoring the lung transcription of inflammatory genes associated with TLR5 signaling and by comparing mRNA levels to animals that received AMX alone. The results showed that doses from 1 to 25 μg per animal saturated the upregulation of transcriptional response for *Cxcl1, Cxcl2, Ccl20, Il1b*, and *Il6* genes. Ultimately, the dose of 2.5 μg of FliC_Δ174−400_ was selected as a saturating immunostimulatory dose in the context of pneumococcal infection and lung inflammation.

### The Combination of Antibiotics and Respiratory Instillation of Flagellin Displays Synergistic Therapeutic Activity Against *S. pneumoniae* Infection

**Figure 2:**
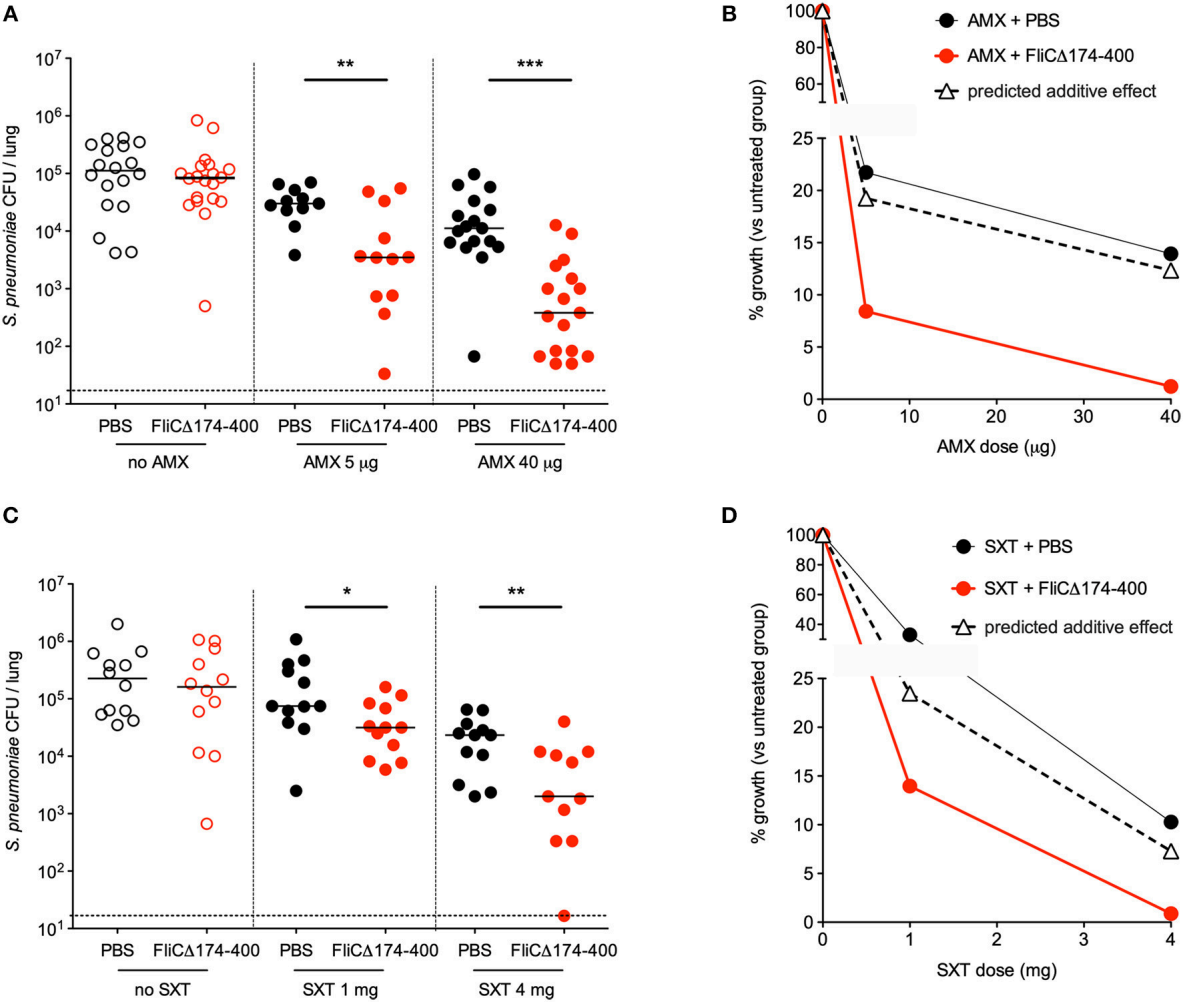
Synergy between intranasal flagellin and antibiotics in the treatment of a pneumococcal lung infection. Swiss mice (*n* = 12–20) were infected intranasally with 4 × 10^6^ pneumococcus Sp1. The animals were treated 12 h later with the intragastric administration of amoxicillin (AMX; 5 or 40 μg) **(A,B)**, the intraperitoneal injection of cotrimoxazole that is the combination of the two antibiotics sulfamethoxazole and trimethoprim (SXT; 1 or 4 mg) **(C,D)**, and the intranasal administration of flagellin FliC_Δ174−400_ (2.5 μg in 30 μl of PBS) or PBS only. Lungs were collected at 12 h post-treatment, homogenized, and plated with serial dilution onto blood agar plates. **(A,C)** Bacterial counts in the lungs of mice. Each symbol represents an individual animal. Colony-forming unit (CFU) counts for individual mice are shown. The solid line represents the median value, and the dashed line represents the detection threshold. Data from flagellin-treated mice were compared with those from PBS-treated mice in a Mann–Whitney test (*p < 0.05, **p < 0.01, and ***p < 0.001). **(B,D)** The treatments' effects on bacterial growth were quantified as the percentage of residual growth (% _growth_) in treated mice (antibiotic + PBS or antibiotic + FliC_Δ174−400_) vs. untreated mice (PBS). The predicted additive effect was calculated as % _growth[antibiotic]_ × % _growth[flagellin]_. The values were plotted according to the dose of antibiotic.

The next set of experiments was designed to characterize the therapeutic interaction between intranasal flagellin FliC_Δ174−400_ and oral AMX. Mice were infected with Sp1 and treated 12 h later with either a single intranasal instillation of flagellin (2.5 μg), a single intragastric administration of suboptimal AMX doses of 5 μg (0.2 mg/kg) or 40 μg (1.6 mg/kg) or the combination treatment. To define the treatments' efficacy, lung bacterial counts were measured at 12 h post-treatment. The results showed that flagellin alone had mostly no antibacterial effect, whereas 5 and 40 μg doses of AMX alone were, respectively, associated with 5- and 7-fold smaller bacterial loads, relative to untreated mice (Figure 2A). The combination treatment (AMX + FliC_Δ174−400_) induced a 10-fold relative decrease in bacterial counts for 5 μg of AMX and a 82-fold relative decrease for 40 μg of AMX—showing that AMX-flagellin combination treatment is more effective than the corresponding dose of AMX or flagellin as monotherapy (Figure 2A). The nature of the interactions between flagellin and antibiotics was further analyzed by comparing the bacterial growth upon treatment. The %_growth_ values for the combination treatment (8.4% for flagellin + AMX 5 μg and 1.2% for flagellin + AMX 40 μg) were much lower than the corresponding predicted %_growth_ values for additive effects, calculated as %_growth[AMX]_ × %_growth[flagellin]_ (19.2% for flagellin + AMX 5 μg and 12.3% for flagellin + AMX 40 μg) (Figure 2B). This experiment indicated strong synergy between the two compounds.

Similar experiments were carried out with the combination of the antibiotic SXT and flagellin (Figures 2C,D). The antibiotic SXT was administered intraperitoneally at doses of 1 and 4 mg (40 and 160 mg/kg, respectively). Flagellin (2.5 μg) significantly improved the therapeutic outcome of SXT treatment, as evidenced by CFU counts in the mice's lungs 12 h after administration of the treatments (Figure 2C). The experimental %_growth_ values for the combination treatment were lower than the corresponding predicted %_growth_ values (14 vs. 23.5% for SXT 1 mg, and 0.88 vs. 7.3% for SXT 4 mg)—reflecting a synergy between flagellin and SXT (Figure 2D).

Taken as a whole, these results show that antibiotics + flagellin had a strong synergistic effect on pneumococcal lung infection in mice. Furthermore, the synergy seems to be independent of the type of antibiotic, since it was observed with a compound that inhibits bacterial cell wall (AMX) and a pair of compounds that inhibits folic acid synthesis (SXT).

### A Model of Pneumonia With Antibiotic-Resistant *S. pneumoniae* in a Post-influenza Context

**Figure 3:**
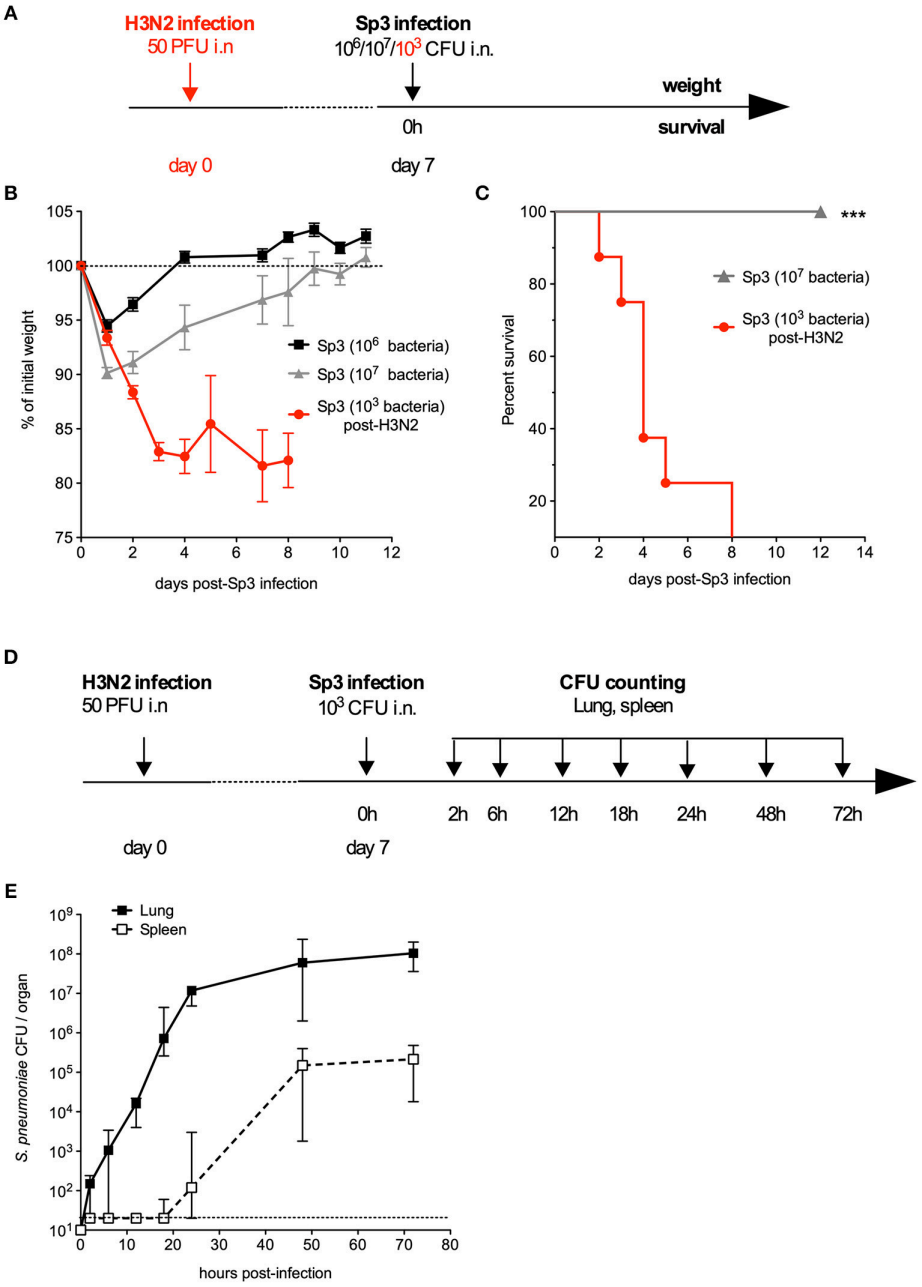
A murine model of pneumonia due to antibiotic-resistant pneumococcus. **(A–C)** C57BL/6J mice (*n* = 5–8) were infected intranasally with 10^6^ or 10^7^ antibiotic-resistant pneumococcus Sp3 in 30 μl of PBS or with 50 PFUs of H3N2 virus in 30 μl of PBS followed 7 days later by intranasal administration of 10^3^ Sp3. **(B)** Body weight was monitored after Sp3 infection and expressed as a percentage of the initial weight. The data are quoted as the mean ± SEM. **(C)** Survival was monitored daily for 12 days. Data were compared in a log-rank test. ***p < 0.001. **(D,E)** C57BL/6J mice were infection intranasally with 50 PFUs of H3N2 virus in 30 μl of PBS followed 7 days later by intranasal administration of 10^3^ Sp3. **(E)** Bacterial counts in the lung and spleen of mice (*n* = 5). Tissues were collected at the indicated times post-Sp3 infection, and plated in serial dilutions on blood-agar plates. The values correspond to the median (range) CFU count. The dashed line represents the detection threshold.

Next, we looked at whether the combination treatment's effect on an antibiotic-sensitive *S. pneumoniae* strain was also exerted on antibiotic-resistant bacteria. To this end, a mouse model of infection with a Sp3 strain that is resistant to a wide range of antibiotics including AMX (MIC_AMX_ = 2 μg/ml, i.e., 125-fold higher than for Sp1) was developed. We found that the Sp3 strain failed to induce a lethal infection and other signs of disease (weight loss) in naïve mice—even at high doses of challenge (10^6^ or 10^7^ bacteria per animal) (Figures 3A,B). Given that the influenza virus infection increases susceptibility to bacterial infections even after it has been eliminated (34–37), Sp3 infection was assessed in mice that had already been exposed to the virus. Briefly, mice were infected first with an intranasal, sublethal dose of H3N2 virus (50 PFU) and then infected 7 days later with 10^3^ CFU of Sp3. This bacterial superinfection induced significant weight loss and was 100% lethal (Figures 3A,B). The bacterial counts increased gradually over time, and reached 10^7^ CFU per lung 24 h post-infection (Figure 3C). Sp3 was also detected in the spleen—indicating a translocation and systemic dissemination of the bacteria—from 24 h post-infection onwards (Figure 3C). In conclusion, the antibiotic-resistant Sp3 strain induced effective pneumonia when animals had been previously exposed to experimental flu.

### An Amoxicilin + Flagellin Combination Is Effective Against Amoxicillin-Resistant *S. pneumoniae*

**Figure 4:**
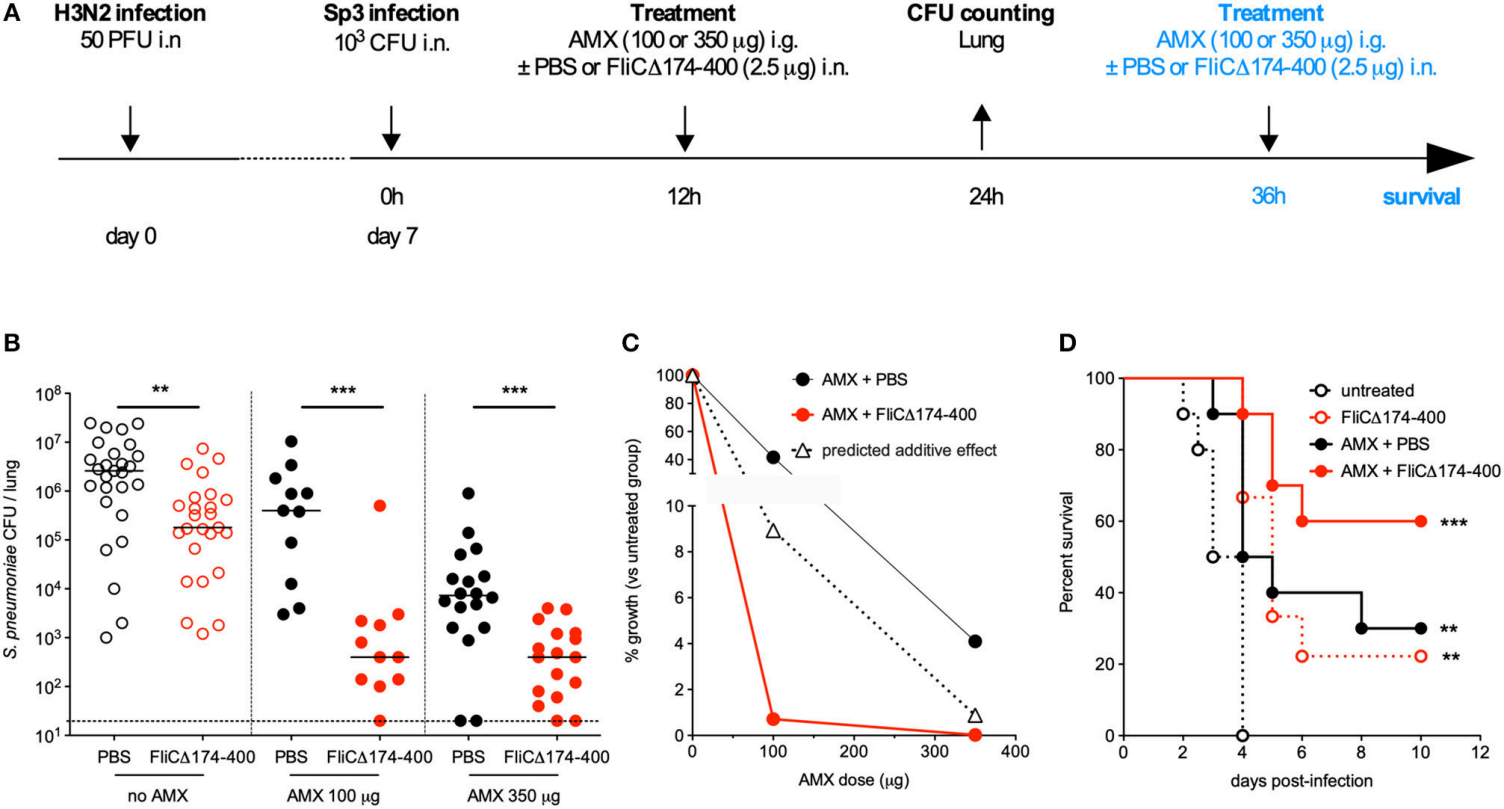
Synergy between amoxicillin and intranasal administration of flagellin in the treatment of pneumonia with antibiotic-resistant pneumococcus. **(A)** C57BL/6J mice (*n* = 12–28) were infected intranasally first with 50 PFUs of H3N2 virus in 30 μl of PBS and then 7 days later with 10^3^ antibiotic-resistant pneumococcus Sp3 in 30 μl of PBS. Mice were treated 12 h after Sp3 infection via the intranasal administration of flagellin FliC_Δ174−400_ (2.5 μg in 30 μl of PBS), the intragastric administration of amoxicillin (AMX; 100 or 350 μg), or combination of both. Lungs were collected 24 h post-infection, homogenized, and plated in serial dilutions onto blood agar plates to measure the bacterial load. For survival experiment, mice received a second dose of the same treatment at 36 h post-Sp3 infection. **(B)** Lung bacterial counts. Colony-forming unit (CFU) counts for individual mice are shown, and the solid line represents the median value. The dashed line represents the detection threshold. Data from flagellin-treated and control (PBS-treated) mice were compared in a Mann-Whitney test (**p < 0.01, and ***p < 0.001). **(C)** The treatments' effects on bacterial growth were quantified as the percentage of residual growth (%_growth_) in treated mice (AMX+PBS or AMX+FliC_Δ174−400_) vs. untreated mice (the PBS group). The predicted additive effect was calculated as %_growth[AMX]_ × %_growth[flagellin]_. The values were plotted according to the dose of AMX. **(D)** Survival was monitored daily for 12 days. Data from the treated groups were compared with data from an untreated group in a log-rank test (**p < 0.01, and ***p < 0.001).

In order to test the efficacy of an antibiotic+flagellin combination treatment in the post-influenza Sp3 superinfection model, mice were treated with AMX alone, flagellin FliC_Δ174−400_ alone, or a combination of both compounds 12 h after the bacterial infection (Figure 4A). Due to the high level of AMX resistance, the doses of antibiotic used were 100 μg (4 mg/kg) and 350 μg (14 mg/kg). Using this regimen, the serum concentration levels of AMX in naïve animals were expected to be close to 1 × MIC and 4 × MIC, respectively (Professor Charlotte Kloft, personal communication). Flagellin treatment alone decreased bacterial counts in the lungs by 5.6-fold, whereas AMX treatments decreased bacterial counts by 3.7-fold (for a dose of 100 μg) and 74.6-fold (for a dose of 350 μg). When AMX was combined with flagellin, bacterial counts were of 5,526- and 5,485-fold lower for the 100 and 350 μg doses of antibiotic, respectively. These results show a significant therapeutic advantage for the combination treatment, relative to standalone AMX or flagellin treatments (Figure 4B). We also determined CFU counts in the spleen; both AMX and AMX + flagellin treatments (either with 100 or 350 μg of the antibiotic) were able to prevent systemic dissemination of the infection (data not shown). Comparison of %_growth_ for the observed effect of the combination treatment vs. predicted additive effect (0.7 vs. 8.9% for AMX 100 μg, and 0.02 vs. 0.9% for AMX 350 μg) demonstrated the synergy of the combination in the context of superinfection and antibiotic resistance (Figure 4C). After two administrations of treatments 12 and 36 h after Sp3 superinfection, the flagellin + AMX combination was found to significantly improve the survival of mice, relative to standalone treatments (Figure 4D). These data strongly suggest that flagellin + AMX have synergistic therapeutic effects to control the antibiotic-resistant pneumococcal infections in relevant pathophysiological contexts.

### The Respiratory Administration of Flagellin During Amoxicillin Treatment Stimulates Innate Immunity in the Context of Pneumococcal Post-influenza Superinfection

**Figure 5:**
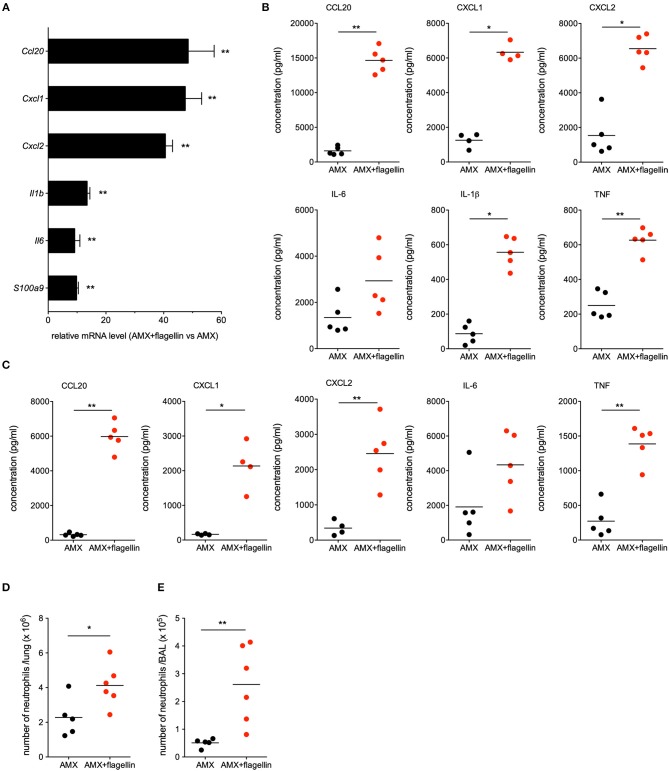
Lung innate immune response during flagellin treatment in post-flu superinfection with antibiotic-resistant pneumococcus. C57BL/6J mice (*n* = 4–6) were infected intranasally first with 50 PFUs of H3N2 virus in 30 μl of PBS and then 7 days later with 10^3^ antibiotic-resistant pneumococcus Sp3 in 30 μl of PBS. Mice were treated 12 h after Sp3 infection with the antibiotic amoxicillin (AMX; 100 μg, intragastric administration) and the intranasal administration of flagellin FliC_Δ174−400_ (2.5 μg in 30 μl of PBS) or PBS only. **(A)** Lungs were collected 2 h after treatment, and homogenized. After RNA extraction, expression levels of selected genes were then analyzed using RT-qPCR assays. The relative expression level for each gene is expressed against that of the reference genes *Actb* and *B2m* and the reference condition AMX+PBS (arbitrarily set to a value of 1). The data represent the mean ± SEM. Lungs **(B)** and BAL fluids **(C)** were collected 6 h after treatment and cytokine and chemokine levels were measured by ELISA. Data from AMX+flagellin-treated and AMX+PBS-treated mice were compared in a Mann-Whitney test and are represented as individual values and mean. Lungs **(D)** and BALs **(E)** were collected 12 h after treatment. Lungs and BAL cell suspensions were stained using a mixture of antibodies specific for surface markers before flow cytometry analysis. Neutrophils were defined as CD45^+^CD11b^+^Ly6G^+^ cells after exclusion of dead cells and alveolar macrophages (CD45^+^SiglecF^+^CD11c^+^ cells) from the analysis. Numbers of neutrophils in the lung parenchyma **(D)** and BAL fluids **(E)** are shown for individual animal and the line represents the mean. Data from AMX+flagellin group were compared to those of AMX+PBS group in a Mann-Whitney test. Statistical significance is indicated as follows: *p < 0.05, and **p < 0.01.

Since infection by influenza virus induces major changes in lung integrity and immune cell populations, we investigated the immunomodulatory impact of flagellin on post-flu respiratory infections by the antibiotic-resistant Sp3 strain. To this end, C57BL/6 mice were infected with influenza A virus at day 0 and then challenged with antibiotic-resistant Sp3 at day 7. Treatments with oral AMX (100 μg) combined or not with intranasal flagellin (2.5 μg) were administered 12 h after Sp3 infection. Lungs were collected 2 h post-treatment for transcriptional analysis using RT-qPCR assays, as described in Figure 1. We observed that despite the superinfection, flagellin still enhanced the transcription of *Cxcl1, Cxcl2, Ccl20, Il1b, Il6*, and *S100a9* genes, i.e., surrogate markers of TLR5-mediated lung stimulation (Figure 5A). We next quantified the cytokine/chemokine production after 6 h of treatment both in the BAL fluids and lung protein extracts. Delivery of flagellin in the lung of AMX-treated pneumococcal superinfection significantly increased levels of CCL20, CXCL1, CXCL2, and Tumor-necrosis factor (TNF) both in the lungs (Figure 5B) and in the BAL fluids (Figure 5C) in AMX+flagellin-treated mice compared with AMX+PBS-treated mice. We also observed increased IL-6 production in both compartments although it was not statistically significant. Production of IL-1β (or pro- IL-1β) was detected only in the lung tissue and was increased in flagellin-treated mice. Finally, we used flow cytometry to evaluate immune cell populations in BAL fluids and lung tissue collected 12 h post-treatment. The analysis showed that the neutrophil counts were higher in mice having receiving the combination treatment (i.e., TLR5 stimulation and AMX) than in mice having receiving AMX alone both in the lung tissues (Figure 5D) and the BAL fluids (Figure 5E). Interestingly, the innate response to combination treatment was also detectable in blood since the production of the inflammatory mediators were significantly augmented at 2 h (for IL-6, CCL20, CXCL1, and CXCL2) and 6 h (CCL20 and CXCL1) compared to AMX alone treatment (Supplementary Figure 1). The blood cytokine production then diminished to an undetectable or very low level at 12 h. Thus, these observations showed that the mucosal delivery of flagellin does not induce sustained systemic inflammation. Overall, the innate immune response to flagellin was effectively stimulated in the context of the influenza immunological imprinting, the superinfection challenge, and the antibiotic treatment.

## Discussion

Our present results demonstrated the synergistic efficacy of a combination of an antibiotic (AMX or SXT) and the local administration of the immunomodulatory biologic flagellin against respiratory infections caused by *S. pneumoniae*. Of note, the efficacy of combined antibiotic + flagellin treatment, previously demonstrated in inbred BALB/c and C57BL/6 mice by Porte et al. (20), was here extended to outbred Swiss mice, showing genetic background independence of the protection. Remarkably, flagellin was able to trigger lung innate immune responses in the context of inflammation (i.e., airways damaged by bacterial pneumonia and flu). Immunostimulation in the lung was a dose-dependent process that was saturating by microgram-per-animal levels of flagellin. The synergy appeared to be independent of the antibiotic dose level and the antibiotic's target, since AMX acts on the bacterial cell wall and SXT inhibits DNA synthesis. The present study is also the first to have demonstrated that stimulating innate immunity can treat severe pneumonia induced by antibiotic-resistant pathogenic bacteria; this may open up new avenues for the treatment of pneumonia in the context of growing antimicrobial resistance.

It has been demonstrated that intranasal administration of flagellin activates TLR5-dependent local innate responses with broad-spectrum antibacterial activity (11, 15, 16, 20, 21, 23). The pulmonary response includes the production of various antimicrobial peptides (i.e., cathelicidin antimicrobial peptide and the β-defensins), cytokines (TNF, IL-1β, and IL-6), and chemokines (i.e., CCL20, CXCL1, CXCL2, CXCL5, and CXCL8). This cytokine and chemokine production is in line with the observed recruitment of phagocytes (and especially neutrophils) in the lung following the intranasal administration of flagellin to naïve mice (15, 23, 38). Flagellin intranasal administration specifically triggers TLR5-mediated transcription in the lungs from 2 to 30 h after a pneumococcus infection or from 7 to 14 days after an influenza infection (20). Here, we demonstrated that the lung innate immune signature induced by intranasal instillation of flagellin is still effective in a highly inflammatory context with associated lung damage (pneumococcal post-influenza superinfection), and is not influenced by antibiotic treatment (Figure 5). Interestingly, earlier reports indicated that influenza infections promote the partial but sustained desensitization of TLR-mediated lung innate responses and a reduction in TLR expression (39). Our observations demonstrate that, in the physiopathological context of superinfection, flagellin is still able to trigger sufficient levels of innate defense and exert synergy with antibiotics (Figure 4).

Airway epithelial cells have been identified as an important component for detection of flagellin and TLR5 signaling at homeostasis (21, 22). These sentinel cells not only sense danger signals introduced in the conducting airways but also produce factors to directly impair the colonization and growth of pathogens or indirectly mobilize phagocytic and immune cells to clear infection. More generally, airway epithelium TLR signaling represent a key driving force in antibacterial defense (40). Recently, Anas et al. demonstrated an essential contribution of epithelial signaling in the respiratory tract in response to flagellin in the context of infection with *Pseudomonas aeruginosa* (41). Our data showed that several antimicrobial peptides (S100A9), cytokines (IL-1β and TNF), and chemokines (CCL20, CXCL1, and CXCL2) that were associated to epithelial responses are also upregulated after the administration of the combination treatment in the post-flu superinfection model, suggesting that the epithelium is also an important flagellin-specific driving force in the lung damaged by viral and bacterial infections. Targeting epithelium is a serious benefit for immunostimulation since it allows reducing the dose and bypassing systemic adverse effects.

Our data contribute to highlight the therapeutic potential of the association of two drugs with distinct modes of action: an antibiotic with a direct effect on bacteria, and a TLR5-specific stimulator of innate immunity with indirect antibacterial activity mobilizing both multiple phagocytic host cells and various antimicrobial factors such as antibacterial peptides, and chemokines and cytokines that mobilize and activate immune cells. Besides pathogen killing, the multitargeting of innate immunity by flagellin could impact on bacterial fitness and thereby increase susceptibility to the antibiotic. The innate immune response induced by TLR5 signaling may also modify the distribution of antibiotic in lung tissues while the antibiotic, by damaging the pathogen, could also enhance the immune signaling. In addition, the pharmacokinetics of the antibiotic and the immunostimulator, i.e., a short-term dose-dependent effect for the antibiotic, and an immediate and long-lasting impact of the immunostimulator due to cell mobilization, are likely complementary. Finally, flagellin, by modulating innate immunity in the respiratory tract, has been shown to enhance the mucosal and systemic adaptive immunity (22, 23). Such property may be of interest to elicit anti-pathogen immune memory and prevent recurrent/relapse infections.

As an opportunistic bacterium, *S. pneumoniae* frequently colonizes the upper respiratory tract and thus represents the prime cause of bacterial-associated CAP (42). However, other microorganisms can cause CAP and healthcare-associated pneumonia; they include Gram-positive bacteria such as *Staphylococcus aureus*, Gram-negative bacteria like *P. aeruginosa, Klebsiella pneumoniae, Haemophilus influenzae*, mycoplasma (*M. pneumoniae*) and intracellular bacteria (*Legionella pneumophila*) (1). The diagnosis and treatment of CAP is complicated by the broad variety of causative agents, and the progression of antibacterial resistance. In this context, immunomodulators such as flagellin are of great interest because they activate a large number of antimicrobial immune mechanisms. Indeed, flagellin has already demonstrated its ability to protect against various pathogens including Gram-negative and Gram-positive bacteria (8, 12–16, 20). Furthermore, our present results showed that the therapeutic synergy between antibiotic and intranasal flagellin is independent of the antibiotic's mechanism of action—suggesting that flagellin can potentially be combined with various antibiotics for a wide range of clinical situations. The synergistic effects of the combined therapy have been determined to be independent of capsule antigenicity (serotype 1 or 3) of pneumococcus, suggesting that the general innate immune protecting mechanisms triggered by flagellin could potentially be effective against a large variety of serotypes.

Given the progression of antibiotic resistance, a model of infection by antibiotic-resistant bacteria would constitute an important tool for developing alternative anti-infectious approaches. We first attempted to develop such a model in immunocompetent animals. The multidrug-resistant clinical isolate of pneumococcus Sp3 was unable to induce a lethal infection, even at high doses. Acquisition of antibiotic resistance is often associated with a loss of bacterial fitness (43), which might explain the Sp3's very low virulence in naïve mice. It is now becoming clear that many cases of bacterial pneumonia result from co-infections or consecutive infections (especially influenza virus infections) (37). As shown by Figures 3D,E, 4, influenza virus infection creates a favorable environment for colonization and invasion by the low-virulence antibiotic-resistant pneumococcus Sp3 strain. Our data demonstrated that the flagellin+AMX combination treatment effectively reduces the bacterial burden caused by the Sp3 strain in the lung, and improves the survival rate among treated mice. Our proof-of-concept findings may be transposable to the clinic for patients with co-infections and superinfections, which are relevant physiopathological causes of hospitalization and complicated pneumonia.

Antibiotics constitute the current standard of care for bacterial pneumonia, and the growing threat of antibiotic resistance is a major public health concern. When defining the dosing regiments of antibiotics used to treat a patient, the physician must take account of the antibiotic' pharmacokinetic and pharmacodynamic characteristics. The relationship between *in vivo* exposure to the drug and *in vitro* susceptibility of the bacteria conditions not only the treatment's clinical outcome (i.e., clearance of the infection) but also adverse effects or drug toxicity (44). Thus, the maximum dose of antibiotic that can be administered to a patient may not be enough to totally clear highly resistant bacteria. Our data suggest that the antibacterial efficacy of these antibiotic dose levels can be synergistically enhanced by the effect of flagellin on lung innate immunity.

Taken as a whole, the present results suggest that the selective boosting of innate lung immunity by flagellin improves the therapeutic outcome of antibiotic treatment. In humans, this approach might be a useful generic alternative to the treatment of bacterial pneumonia, thereby reducing the antibiotic dose and regimen as well as the emergence of antibiotic resistance. Moreover, such strategy promotes multitarget inhibition through multiple innate immune effectors that should be more resistant to the development of resistance and may restore some antibacterial activity to antibiotic in the context of antibiotic resistance. Characterization of flagellin's contribution to the lung antibacterial defenses at the molecular and cellular level and the protein's synergy with antibiotics is likely to open up new avenues for the immunotherapy of respiratory tract infections.

## Ethics Statement

All experiments complied with institutional regulations and ethical guidelines (C59-350009, Institut Pasteur de Lille; Protocol Apafis #5164 - 2015121722429127). The protocols were validated by the ethical committee for animal experiments (Comitéd'éthique en expérimentation animale—Nord-Pas-de-Calais CEEA 75).

## Author Contributions

LM performed all animal, RT-qPCR, and flow cytometry experiments. FC, RP, DC, and CF provided LM with technical assistance. FW analyzed the bacterial species and antibiotic resistance. LM, CC, and J-CS designed experiments and wrote the manuscript. J-CS and CC supervised the experimental work as a whole.

### Conflict of Interest Statement

The authors declare that the research was conducted in the absence of any commercial or financial relationships that could be construed as a potential conflict of interest.
