# Supplementary figures and images for: Therapeutic Synergy Between Antibiotics and Pulmonary Toll-Like Receptor 5 Stimulation in Antibiotic-Sensitive or -Resistant Pneumonia
Source: Front Immunol. 2019 Apr 9;10:723. doi: 10.3389/fimmu.2019.00723 (PMC6465676; doi:10.3389/fimmu.2019.00723)

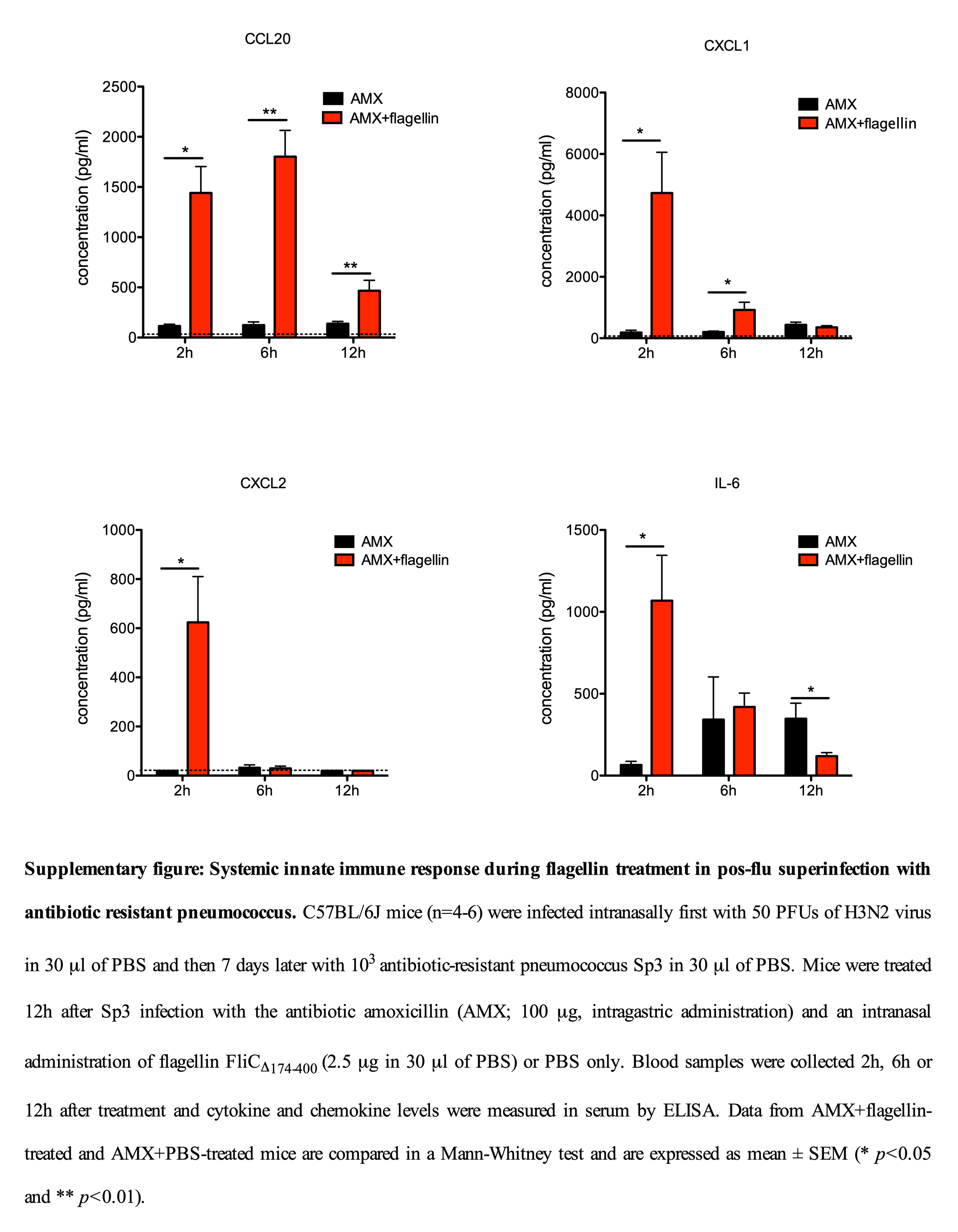

Supplement: Supplementary file 1 [file Image_1.TIFF]
